# Diagnostic performance of a high-spatial-resolution voxelwise analysis of neuromelanin-sensitive imaging in early-stage idiopathic Parkinson’s disease

**DOI:** 10.1186/s12880-023-01018-1

**Published:** 2023-05-18

**Authors:** Minjung Seong, Seongbeom Park, Young Hee Sung, Eung Yeop Kim

**Affiliations:** 1 Department of Radiology, Samsung Medical Center, Sungkyunkwan University School of Medicine, 81, Irwon-ro, Gangnam-gu, Seoul, 06351 Republic of Korea; 2 Heuron Co., Ltd, Incheon, Republic of Korea; 3 Department of Neurology, Gil Medical Center, Gachon University College of Medicine, Incheon, Republic of Korea

**Keywords:** Idiopathic Parkinson’s disease, substantia nigra, Neuromelanin-sensitive MRI, Voxelwise analysis, Contrast ratio

## Abstract

**Background:**

Quantitative assessments of neuromelanin (NM) of the substantia nigra pars compacta (SNpc) in neuromelanin-sensitive MRI (NM-MRI) to determine its abnormality have been conducted by measuring either the volume or contrast ratio (CR) of the SNpc. A recent study determined the regions in the SNpc that are significantly different between early-stage idiopathic Parkinson’s disease (IPD) patients and healthy controls (HCs) using a high spatial-resolution NM-MRI template, which enables a template-based voxelwise analysis to overcome the susceptibility of CR measurement to inter-rater discrepancy. We aimed to assess the diagnostic performance, which has not been reported, of the CRs between early-stage IPD patients and HCs using a NM-MRI template.

**Methods:**

We retrospectively enrolled early-stage IPD patients (n = 50) and HCs (n = 50) who underwent 0.8-mm isovoxel NM-MRI and dopamine-transporter PET as the standard of reference. A template-based voxelwise analysis revealed two regions in nigrosomes 1 and 2 (N1 and N2, respectively), with significant differences in each substantia nigra (SNpc) between IPD and HCs. The mean CR values of N1, N2, volume-weighted mean of N1 and N2 (N1 + N2), and whole SNpc on each side were compared between IPD and HC using the independent t-test or the Mann-Whitney U test. The diagnostic performance was compared in each region using receiver operating characteristic curves.

**Results:**

The mean CR values in the right N1 (0.149459 vs. 0.194505), left N1 (0.133328 vs. 0.169160), right N2 (0.230245 vs. 0.278181), left N2 (0.235784 vs. 0.314169), right N1 + N2 (0.155322 vs. 0.278143), left N1 + N2 (0.140991 vs. 0.276755), right whole SNpc (0.131397 vs. 0.141422), and left whole SNpc (0.127099 vs. 0.137873) significantly differed between IPD patients and HCs (all *p* < 0.001). The areas under the curve of the left N1 + N2, right N1 + N2, left N1, right N1, left N2, right N2, left whole SNpc, and right whole SNpc were 0.994 (sensitivity, 98.0%; specificity, 94.0%), 0.985, 0.804, 0.802, 0.777, 0.766, 0.632, and 0.606, respectively.

**Conclusion:**

Our NM-MRI template-based CR measurements revealed significant differences between early-stage IPD patients and HCs. The CR values of the left N1 + N2 demonstrated the highest diagnostic performance.

**Supplementary Information:**

The online version contains supplementary material available at 10.1186/s12880-023-01018-1.

## Introduction

Since the first application of neuromelanin-sensitive MRI (NM-MRI) to idiopathic Parkinson’s disease (IPD) [1], many studies have been conducted using NM-MRI in patients with parkinsonism. In fact, the validity of using NM-MRI to assess NM in the substantia nigra pars compacta (SNpc) has been proven in a recent study in which the signal intensity in the SNpc on NM-MRI is correlated with its NM concentration [2]. Quantitative assessments of NM of the SNpc in NM-MRI to determine its abnormality have been conducted by measuring either the volume or contrast ratio (CR) of the SNpc.

Volume measurement of the SNpc on NM-MRI is performed by segmentation using a prespecified threshold to calculate the whole or regional volume of the SNpc [3–20]. Although segmentation is mostly performed in a semi-automated fashion, inter-rater discrepancy can occur when determining the reference region(s). Volume measurements may also be cumbersome in that corrections should be performed by measuring the intracranial volume in addition to measuring the SNpc volume of each individual. Finally, the SNpc in patients with nigrostriatal degeneration tends to lose its hyperintensity. [1, 3, 4] These diseased SNpc may have ambiguous boundaries, which could, consequently, increase the inter-rater discrepancy.

In a recent study that compared the SNpc volume of de novo IPD and the control group using a 0.9 mm-thick magnetization transfer-prepared 3T MRI, the area under the curve (AUC) value was 0.82 [19], implying that the diagnostic value of volume measurement is not yet high enough between de novo IPD patients and healthy controls (HCs). A deep learning-based approach using a convolutional neural network may be a good alternative way to measure the volumes of the SNpc in NM-MRI. Using a U-net, a recent study showed high accuracy [7]. However, they included patients with advanced-stage IPD, which may overstate the diagnostic performance. In fact, it would be more clinically relevant to assess only patients with early-stage IPD, but results with patients with early-stage IPD using a more objective method are still limited.

CR measurement is a method of measuring the signal intensity ratio between the two or three user-defined subdivisions of the SNpc and the reference region [21–25]. This method may be more susceptible to inter-rater discrepancy than volume measurement for several reasons. First, manual drawing of the ROI is inevitably vulnerable to inter-rater discrepancies. Second, the significantly affected regions of the SNpc tend to lose their hyperintensity, and consequently, they may be selected only in part or not at all when defining the SNpc for CR measurement. Instead of manually drawing of ROIs in the SNpc of each subject, it would be desirable to use the ROIs that are determined by a template-based voxelwise analysis. A recent study determined the regions in the SNpc that are significantly different between early-stage IPD patients and HCs using a high spatial-resolution NM-MRI template [26]. In this study, however, the authors did not show the diagnostic performance of the CR values of the areas with significant difference. We hypothesized that the CR measurement of these areas in the NM-MRI template may show high diagnostic accuracy. The purpose of this study was therefore to assess the diagnostic performance of CR measurements using an NM-MRI template.

## Materials and methods

### Participants

In this retrospective study approved by the institutional review board, 50 consecutive patients who were diagnosed with de novo IPD in the period between January 2018 and October 2019 in accordance with the Movement Disorder Society clinical diagnostic criteria for Parkinson’s disease [27] were enrolled. All patients underwent N-3-fluoropropyl-2-b-carbomethoxy-3-b-(4-iodophenyl) nortropane PET (18 F-FP-CIT PET), which served as the standard of reference. All 50 patients with IPD were drug-naïve. Motor symptoms were assessed using the Hoehn and Yahr scales [28] and Unified Parkinson’s Disease Rating Scale (UPDRS). The clinical laterality of the motor symptoms was evaluated by using the UPDRS III scores. Scores of resting tremor, rigidity, finger tapping, hand movement, and rapid alternative movements of hand and leg agility were summed in the right and left sides, respectively. When the score of one side was higher than that of the other side by two points, the higher score side was determined as the dominant side.

Fifty healthy age- and sex-matched subjects were enrolled as the control group. Individuals without known neurological diseases (e.g., any type of dementia, movement disorders, or stroke), subjective memory complaints, or objective cognitive rine were recruited via local advertisement [29], and participants who exhibited significant structural abnormalities on MRI were excluded. All participants had Clinical Dementia Rating scores of 0 and normal results on neuropsychological tests. The demographics and clinical characteristics of the study population are summarized in Table 1.

**Table 1:** Demographics and clinical characteristics of the study population

	IPD (n = 50)	Healthy subjects (n = 50)	*p* value
Age (years)	69.0 (58.3–78.0)	67 (63.3–71.8)	0.320
Sex (Male:Female)	23:27	16:34	0.373
Disease duration (months)*	6 (3.3–12.0)	–	–
Hoehn and Yahr stage	2 (1–2)	–	–
UPDRS I	0 (0–2)	–	–
UPDRS II	6 (4–7)	–	–
UPDRS IIII	13 (9–21)	–	–
Handedness (Right:Left)	45:5	47:3	1.000

Note: Data are the median, with interquartile ranges in parentheses

IPD, idiopathic Parkinson’s disease; UPDRS, Unified Parkinson’s Disease Rating Scale

* Time since symptom onset

The enrolled participants were also assessed in a previous study [26]. However, this previous study did not assess the diagnostic performance of the regions that were determined by an NM-MRI template.

### Image acquisition

All participants underwent MR imaging using a 3 T scanner with a 32-channel coil (Skyra; Siemens Healthineers, Forchheim, Germany). Whole-brain sagittal 3D magnetization prepared rapid gradient-echo imaging, oblique axial 3D multi-echo gradient-recalled echo (GRE) imaging, and 3D T1-weighted sampling perfection with application optimized contrast using different flip-angle evolution (SPACE) imaging with delay alternating with nutation for tailored excitation (DANTE) preparation were obtained. DANTE was applied to improve the delineation of the SNpc [30]. Both 3D GRE and 3D SPACE with DANTE were obtained in the same oblique axial plane parallel to the plane from the posterior commissure and top of the pons. The detailed parameters are summarized in the Online Resource Table S1. Quantitative susceptibility mapping (QSM) using both magnitude and phase images of multi-echo GRE imaging and susceptibility map-weighted imaging (SMwI) using QSM as a mask were sequentially reconstructed [31, 32].

18 F-FP-CIT PET in the 3D scanning mode (3-mm thick, 40 slices) was obtained using PET/CT scanner (Biograph-6; Siemens, Erlangen, Germany).

### Voxelwise contrast ratio analysis using a template

**Fig. 1:**
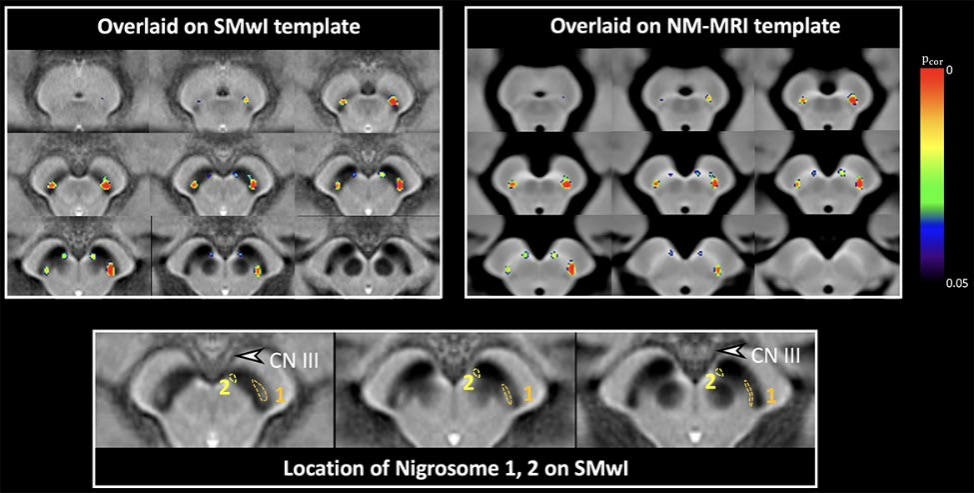
All images are resampled perpendicular to the midbrain axis. Two separate areas with significant statistical difference (a corrected *p* value less than 0.05) in each side of the substantia nigra (overlaid on SMwI [Top left corner] and NM-MRI templates [Top right corner]) were determined through a voxelwise analysis of neuromelanin-sensitive imaging between early-stage IPD patients and healthy subjects. The presumed nigrosome 1 and 2 regions are defined on SMwI template (bottom row). This figure is modified from the Figs. 2 and 3 of the previous article [26]

The templates to determine the significantly affected regions of the SNpc in early-stage IPD when compared to that in HCs through voxelwise analysis were created by preprocessing of the NM-MRI and SMwI. NM-MRI and SMwI were normalized in terms of space and intensity, followed by skull-stripping. Then NM-MRI and SMwI were rigidly registered to each other using Advanced Normalization Tools (ANTs; https://www.nitrc.org/projects/ants). The normalized images are averaged to create a template. The details of creating templates are described elsewhere (Online Resource) [26]. Two regions in each SNpc showing significant differences between early-stage IPD patients and HCs were determined (Fig. 1). The larger region was located in the posterolateral aspect of the SNpc, and was presumably in nigrosome 1, and we thus designated this region as N1. The smaller region was found in the anteromedial region of the SNpc, which was presumed to be in nigrosome 2, and the ROI was thus named N2. The CR values of N1 and N2 on each side were measured for all subjects, and the volume of each ROI was measured. The volume-weighted mean of CR values of N1 and N2 on each side, defined as the volume-weighted mean, was calculated according to the formula:

$$$$$$$$$$\begin{array}{l}Volume - wasted\,mean\\= \frac{{\left( {C{R_{N1}} \times are{a_{N1}} + C{r_{N2}} \times are{a_{N2}}} \right)}}{{are{a_{N1}} + are{a_{N2}}}}\end{array}$$

The whole SNpc was manually defined on the NM-MRI template by a neuroradiologist with 21 years of experience (E.Y.K.). The CR values of the entire SNpc on each side were also measured.

The mean CR values were compared between IPD patients and HCs by using the independent t-test or the Mann-Whitney U test, depending on the results of tests of normality and Bonferroni correction was conducted to counteract multiple comparisons. The diagnostic performance was compared in each region using the receiver operating characteristic curves. The N1 region, which has a lower CR value for each subject, was determined. We tested whether clinical laterality was concordant with the side of a higher CR value in each patient (e.g., in a patient with left clinical laterality, the CR value was higher in the left SNpc), using the Fisher’s exact test. Internal validation about the AUC was performed using leave-one-out cross validation.

Statistical significance was set at *p* < 0.05. Statistical analyses were conducted using SPSS (version 27, Chicago, IL, USA), MedCalc version 20.009 (MedCalc Software, Mariakerke, Belgium), and R 4.0.3 (Vienna, Austria; http://www.R-project.org/).

## Results

The volume of the left N1 (42.750 mm^3^) was greater than that of the right N1 (14.375 mm^3^). There was also asymmetry in the volume of N2 on each side (2.625 mm^3^ and 1.125 mm^3^ on the left and right sides, respectively).

**Fig. 2:**
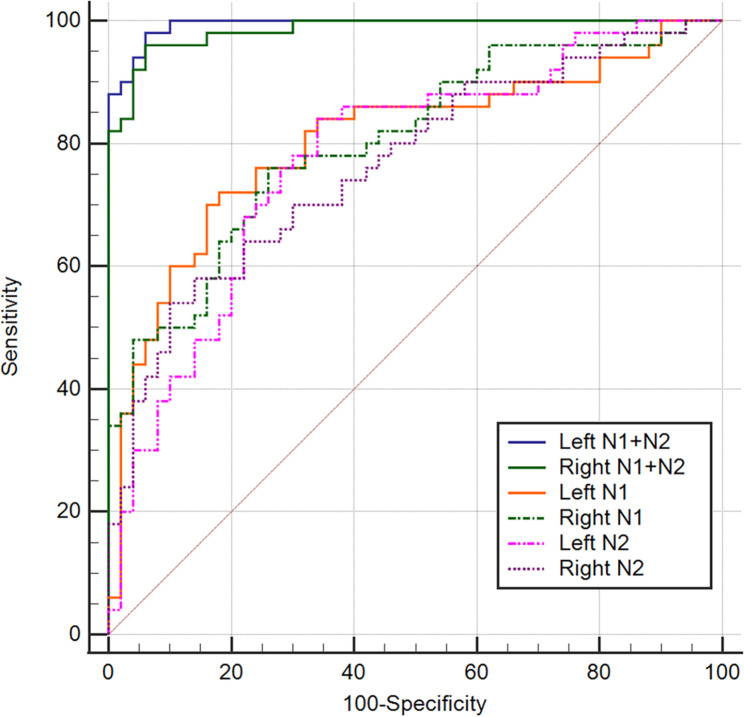
Pairwise comparison of receiver operating characteristic curves N1, the region of interest in nigrosome 1; N2, the region of interest in nigrosome 2; N1 + N2, volume-weighted mean of N1 and N2

**Fig. 3:**
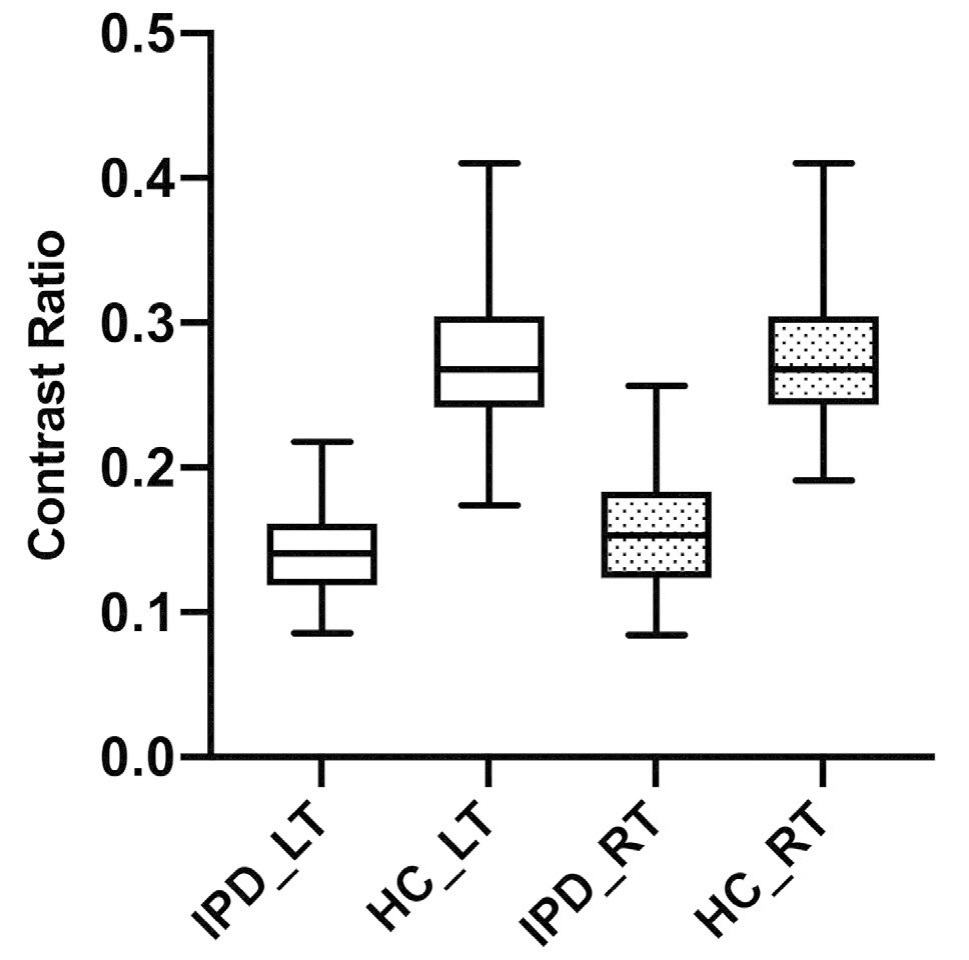
Box and whisker plots for the contrast ratio values of the volume-weighted mean of the regions in nigrosome 1 and nigrosome 2 on both sides between patients with idiopathic Parkinson’s disease (IPD) and healthy controls (HCs). RT, right; LT, left

We found significant differences in the CR values in N1 and N2, volume-weighted mean of N1 and N2 (N1 + N2), and the whole SNpc between IPD patients and HCs (all *P* < 0.001) (Table 2). The areas under the curve of the mean CR values of left N1 + N2, right N1 + N2, left N1, right N1, left N2, right N2, left whole SNpc, and right whole SNpc were 0.994 (95% CI, 0.952–1.000), 0.985 (95% CI, 0.937–0.999), 0.804 (95% CI, 0.712–0.876), 0.802 (95% CI, 0.711–0.875), 0.777 (95% CI, 0.683–0.854), 0.766 (95% CI, 0.671–0.845), 0.632 (95% CI, 0.529–0.726), and 0.606 (95% CI, 0.503–0.702), respectively (Fig. 2). The result of leave-one-out cross validation is included in the Online Resource (Table S2). The diagnostic performance when using the mean CR values of left N1 + N2 (sensitivity of 98% and specificity of 94%) and right N1 + N2 (sensitivity of 96% and specificity of 94%) were significantly better than those found when using any other individual subregion (*p* < 0.001) (Table 3). Box and whisker plots are given for the CR values of N1 + N2 on both sides between patients with IPD and HCs (Fig. 3). The diagnostic performance of the left N1 + N2 versus right N1 + N2 did not show a significant difference (*p* > 0.999).

**Table 2:** Comparison of contrast ratio values of N1, N2, volume-weighted mean of N1 and N2, and whole SNpc in IPD patients and healthy control subjects

	Contrast Ratio Values^‡^		*p* value§
	IPD (n = 50)	Healthy Control (n = 50)	
Right N1	0.149459 ± 0.038455	0.194505 ± 0.037177	< 0.001*
Left N1	0.133328 ± 0.032071	0.169160 ± 0.031976	< 0.001*
Right N2	0.230245 ± 0.046347	0.278181 ± 0.047634	< 0.001*
Left N2	0.235784 ± 0.043828	0.314169 ± 0.048772	< 0.001*
Right N1 + N2	0.153235 (0.123855–0.179959)	0.267800 (0.243768–0.303305)	< 0.001^†^
Left N1 + N2	0.140459 (0.118907–0.159236)	0.267800 (0.242878–0.303305)	< 0.001^†^
Right whole SNpc	0.131397 ± 0.026681	0.141422 ± 0.024871	< 0.001*
Left whole SNpc	0.127099 ± 0.024357	0.137873 ± 0.024597	< 0.001*

Note. IPD, idiopathic Parkinson’s disease; N1, the region of interest in the presumed nigrosome 1 region; N2, the region of interest in the presumed nigrosome 2 region; N1 + N2, volume-weighted mean of N1 and N2; SNpc, the substantia nigra pars compacta

* Independent sample t-test

^†^ Mann-Whitney U test

^‡^ Contrast ratio values are presented as the mean ± standard deviation, except for the right and left N1 + N2, which are presented as the median (interquartile range)

§ Adjusted by Bonferroni correction

**Table 3:** Pairwise comparison of receiver operating characteristic curves

	Left N1	Right N1	Left N2	Right N2	Left N1 + N2	Right N1 + N2	Left whole SNpc	Right whole SNpc
Left N1								
Right N1	> 0.999							
Left N2	> 0.999	> 0.999						
Right N2	> 0.999	> 0.999	> 0.999					
Left N1 + N2	< 0.001	< 0.001	< 0.001	< 0.001				
Right N1 + N2	< 0.001	< 0.001	< 0.001	< 0.001	> 0.999			
Left whole SNpc	< 0.001	< 0.001	0.210	0.336	< 0.001	< 0.001		
Right whole SNpc	< 0.001	< 0.001	0.063	< 0.001	< 0.001	< 0.001	> 0.999	

Note. The presented numbers are *p* values adjusted by Bonferroni correction. N1, the region of interest in the nigrosome 1; N2, the region of interest in the nigrosome 2; N1 + N2, volume-weighted mean of N1 and N2; SNpc, the substantia nigra pars compacta

Clinical laterality was noted on the right in 38 patients, whereas a right N1 with a higher CR value was observed in 30 patients, indicating that both were significantly associated (*p* = 0.027).

## Discussion

In this study, we analyzed the diagnostic performance of the CR measurement in SNpc on NM-MRI utilizing the template created by normalizing and transforming the acquired NM-MRI and SMWI. The result shows significant differences between the IPD patients and HCs in each N1, N2, N1 + N2, and whole SNpc (p < 0.001).

Given the size and shape of the SNpc, it would be desirable to obtain NM-MRI at the plane perpendicular to the midbrain axis [33]. Slice thickness is also important when considering the height of the SNpc (~ 15 mm). In fact, Wengler et al. recently showed that it is more reliable to assess the SNpc using NM-MRI data with 1.5 mm or thinner slice-thickness than using those with thicker slices [34]. Previous studies have used NM-MRI with various slice thicknesses (ranging from 1 to 3 mm), and most obtained relatively thick (> 1.5 mm) NM-MRI parallel to the anterior commissure–posterior commissure (AC-PC) plane [35]. Such thicker NM-MRI parallel to the AC-PC plane may be more susceptible to the partial volume effect, making it less reliable when measuring the volume or CR of the SNpc. The high accuracy in this study may be attributable to the relatively higher spatial-resolution NM-MRI (0.8-mm isovoxel).

The SNpcs of the HCs are relatively well demarcated compared to those of IPD patients. There are the areas of decreased T1 hyperintensity in the SNpc in patients with IPD, which may not be selected for measuring the CR of the SNpc. In this regard, it could be suggested that the volume measurement of the SNpc may outperform the CR measurement of the SNpc for diagnostic purposes. To overcome this limitation of CR measurement, we adopted a template-based approach to measure the CR of the SNpc, which also helped us to minimize the interobserver discrepancy. This unique method may help yield a higher diagnostic performance (AUC, 0.994, which was the best) in our study with de novo IPD patients when compared to the results (AUC, 0.82) of a recent volumetric study with de novo IPD patients [19]. In addition to diagnostic purposes, the CR measurement method using a template may be more appropriate for a longitudinal study design.

In addition to measuring the CR values of the whole SNpc on NM-MRI, we measured CR values in two specific regions of each SNpc, which were found through a voxelwise analysis between de novo IPD patients and HCs, and their locations were determined in the nigrosome 1 and nigrosome 2 regions using the SMwI template [26]. The CR values of these two regions showed a relatively good diagnostic performance between IPD patients and HCs (AUC values ranged from 0.766 to 0.804). However, N1 + N2 in conjunction showed even better diagnostic performance than N1 or N2 alone (left and right AUC, 0.994 and 0.985, respectively). Although it is uncertain why this measurement showed a higher diagnostic performance, it may be explained by the fact that nigrosome 1 is known to be the most severely affected region in patients with IPD, followed by nigrosome 2 [36]. Thus, our results suggest that CR measurement may not be focused only on the posterolateral region of the SNpc.

In previous studies that analyzed CR by dividing the SNpc into two or three subregions, the CR decrease was more prominent in the lateral part of the SNpc in the PD group than in the HC group in most reports [21–23, 37]. However, there have also been reports that CR reduction was statistically significant in all two or three subregions, although there has been no study analyzing the SNpc as a whole [24, 25]. In older studies that did not analyze the SNpc by dividing it into subregions [1, 14], the CR of the whole SNpc was not measured, but the CR values of the circular ROIs drawn in the hyperintense SNpc were assessed. In these studies, the CR of each circular ROI was not assessed separately. In our study, we measured the CR of the whole SNpc as well as N1 and N2 using a template, and found that the diagnostic performance of CR measurement of the whole SNpc was lower than that of N1, N2, and the N1 + N2.

In this study, our best results were obtained when using the mean of the left N1 and N2: sensitivity, 98%; specificity, 94%. These results are more accurate than those in a recent meta-analysis, where the pooled sensitivity was 88% and the pooled specificity was 73% based on the signal intensity [35]. As for the volume measurement, this meta-analysis revealed a pooled sensitivity of 88% and pooled specificity of 84%, which were also lower than ours. In this meta-analysis study, subgroup analysis was additionally performed by dividing the study subjects into disease duration groups ≥ 5 years and < 5 years. As a result, specificity was found to increase when the disease duration was ≥ 5 years. When dividing the groups by 10 years, both sensitivity and specificity were found to increase in the group with a disease duration of 10 years or longer. Given that the median disease duration of the patients in our study was 6 months, the high diagnostic performance of our study may have clinical implications.

This study has some limitations. First, it was a single-center, retrospective study. However, the high-spatial-resolution template used in our study can be used in other studies. This is a preliminary study that shows that the voxelwise analysis using a template is effective in discriminating between PD and HC. Further studies with larger sample sizes and prospective multicenter studies are warranted. Second, the study population was relatively small. Nevertheless, we were able to enroll only de novo IPD patients with a median disease duration of 6 months. Lastly, we did not have pathological confirmation of the results of the SNpc. However, conducting autopsies in such patients with early-stage IPD is extremely difficult. Instead, all patients in our study underwent 18 F-FP-CIT PET, which helped exclude participants without nigrostriatal degeneration.

## Conclusions

Our NM-MRI template-based measurements of CR in the SNpc showed significant differences between patients with early-stage IPD and HCs. The mean of the CR values of the left N1 and N2 demonstrated the highest diagnostic performance.

## Electronic supplementary material

Below is the link to the electronic supplementary material.

Supplementary Material 1

## Data Availability

The datasets analyzed during the current study are not publicly available due to patients’ privacy but are available from the corresponding author upon reasonable request.

## Abbreviations

NM-MRI: Neuromelanin-sensitive MRI
IPD: Idiopathic Parkinson’s disease
SNpc: Substantia nigra pars compacta
CR: Contrast ratio
AUC: Area under the curve
18F-FP-CIT: N-3-fluoropropyl-2-β-carbomethoxy-3-β-(4-iodophenyl)nortropane
SPACE: Sampling perfection with application optimized contrast using different flip-angle evolution
DANTE: Delay alternating with nutation for tailored excitation
QSM: Quantitative susceptibility mapping
SMwI: Susceptibility map-weighted imaging
N1: A region in nigrosome 1 with statistically significant differences between patients with idiopathic Parkinson’s disease and healthy controls
N2: A region in nigrosome 2 with statistically significant differences between patients with idiopathic Parkinson’s disease and healthy controls
N1 + N2: Volume-weighted mean of N1 and N2

## Code Availability

All the data, materials and software application support our published claims and comply with field standards.
