# Supplementary material for: Diagnostic performance of a high-spatial-resolution voxelwise analysis of neuromelanin-sensitive imaging in early-stage idiopathic Parkinson’s disease
Source: BMC Med Imaging. 2023 May 18;23:64. doi: 10.1186/s12880-023-01018-1 (PMC10193688; doi:10.1186/s12880-023-01018-1)
Supplement: Supplementary file 1 — Supplementary Material 1 [file 12880_2023_1018_MOESM1_ESM.docx]

### Online Resource

### Preprocessing and creating templates

The neuromelanin-sensitive MRI (NM-MRI) template was used to determine the significantly affected regions of the substantia nigra pars compacta (SNpc) in patients with early-stage idiopathic Parkinson’s disease (IPD) when compared to healthy controls. The high-spatial-resolution susceptibility map-weighted imaging (SMwI) template was employed to localize these areas in each nigrosome, as SMwI provides anatomical information on nigrosomes, while NM-MRI does not. Using the nonparametric nonuniform intensity normalization algorithm, the nonuniform artifacts of all MRIs were corrected [1]. Thereafter, non-brain tissues were removed using an automated segmenting method, the Brain Extraction Tool [2]. The skull-stripped images were registered linearly to a common coordinate space using version 2.1.52–0 of the Advanced Normalization Tools (ANTs; https://www.nitrc.org/projects/ants), which provided a linear transform matrix. Both NM-MRI and SMwI were rigidly co-registered to their corresponding individual MRIs. The matrices obtained from the two above registration processes were concatenated and applied to both native NM-MRI and SMwI.

The signal intensity of the pre-processed images was normalized and averaged to create the initial target template, followed by the transformation of the new target template with the average affine transformation matrix and the average inverse warp field. These steps were iterated twenty times, and a symmetric template was built using the method of Fonov et al [3].

Spatial normalization of all NM-MRI images was performed using the NM-MRI template to calculate the contrast ratio (CR). Two background regions (BG) of interest (each 3.5-mm sphere) were drawn on each side of the cerebral peduncles on the NM-MRI template. The NM-MRI CR for each voxel (V) was calculated as the relative change in NM-MRI signal intensity (SI) with respect to the BG.

$$\mathrm{CR}_{v}=\frac{\left( \mathrm{SI}_{v}-SI_{\mathrm{BG}} \right)}{SI_{\mathrm{BG}}}$$

Voxelwise analysis started with spatial normalization of all NM-MRI data from patients with IPD to the 0.5 mm isovoxel resampled NM-MRI template with a skull mask. Spatial smoothing of the images with a 1.5-mm full-width-at-half-maximum Gaussian kernel was applied. Sequentially, an inclusive mask of the SNpc was created by manual tracing on the NM-MRI template. Permutation-based non-parametric tests (10,000 permutations) were conducted using the Randomize tool in the FMRIB Software Library (FSL) to assess the spatial differences between healthy subjects and patients with IPD. Threshold-free cluster enhancement (TFCE) in FSL was employed to find clusters in the data (*P* < 0.05, TFCE-corrected). Postprocessing of SMwI data from patients with IPD was implemented in a similar manner.

References

1. Sled JG, Zijdenbos AP, Evans AC (1998) A nonparametric method for automatic correction of intensity nonuniformity in mri data. IEEE Trans Med Imaging 17:87-97 DOI: 10.1109/42.668698.

2. Smith SM (2002) Fast robust automated brain extraction. Hum Brain Mapp 17:143-155 DOI: 10.1002/hbm.10062.

3. Fonov V, Evans AC, Botteron K, Almli CR, McKinstry RC, Collins DL, Brain Development Cooperative G (2011) Unbiased average age-appropriate atlases for pediatric studies. Neuroimage 54:313-327 DOI: 10.1016/j.neuroimage.2010.07.033.

Table S1.

MRI parameters

| Parameters | MP-RAGE | 3D multi-echo GRE | 3D SPACE with DANTE |
| --- | --- | --- | --- |
| Repetition time | 1800 ms | 48 ms | 900 ms |
| Echo time | 3 ms | 14.38 ms (minimum) | 4.8 ms |
| Inversion time | 920 ms |  |  |
| Echo train length |  | 3 |  |
| Echo train duration |  |  | 338 ms |
| Echo spacing |  | 12.3 ms | 4.76 ms |
| Matrix | 256 × 256 | 384 × 384 | 288 × 288 |
| Field of view | 250 × 250 | 192 × 192 (100% phase resolution) | 230 × 230 |
| Slice thickness |  | 1 mm | 0.8 mm |
| Slice number |  | 32 | 208 |
| Acceleration factor | 2* | 2* | 2^†^ |
| Acquisition time | 3 min 35 s | 4 min 46 s | 5 min 12 s |

Note. MP-RAGE (magnetization-prepared rapid gradient-echo); GRE, gradient-recalled echo; SPACE (sampling perfection with application optimized contrast using different flip-angle evolution); DANTE (delay alternating with nutation for tailored excitation); *GRAPPA (generalized autocalibrating partial parallel acquisition); ^†^CAIPIRINHA (controlled aliasing in parallel imaging results in higher acceleration)

Table S2. Results of leave-one-out cross validation

|  | Left N1+N2 | Right N1+N2 | Left N1 | Right N1 | Left N2 | Right N2 | Left whole SNpc | Right whole SNpc |
| --- | --- | --- | --- | --- | --- | --- | --- | --- |
| AUC | 0.990 | 0.976 | 0.787 | 0.789 | 0.757 | 0.750 | 0.603 | 0.560 |
| 95% confidence interval | 0.978-1.000 | 0.951-1.000 | 0.694-0.880 | 0.701-0.878 | 0.661-0.854 | 0.654-0.846 | 0.491-0.715 | 0.446-0.675 |
